# Exploring stigma associated with mental health conditions and alcohol and other drug use among people from migrant and ethnic minority backgrounds: a protocol for a systematic review of qualitative studies

**DOI:** 10.1186/s13643-021-01875-3

**Published:** 2022-01-18

**Authors:** Caitlin H. Douglass, Megan S. C. Lim, Karen Block, Gerald Onsando, Margaret Hellard, Peter Higgs, Charles Livingstone, Danielle Horyniak

**Affiliations:** 1 Burnet Institute, Melbourne, Victoria Australia; 2 Melbourne School of Population and Global Health, University of Melbourne, Melbourne, Victoria Australia; 3 School of Public Health and Preventive Medicine, Monash University, Melbourne, Victoria Australia; 4 Melbourne School of Social and Political Sciences, University of Melbourne, Melbourne, Victoria Australia; 5 Public Health Department, La Trobe University, Bundoora, Victoria Australia

**Keywords:** Stigma, Discrimination, Mental health, Substance use, Migrants, Ethnic groups, Alcohol, Drugs

## Abstract

**Background:**

Stigma is a social process that impedes access to support for mental health conditions and alcohol and other drug (AOD) use, particularly for people from migrant and ethnic minority backgrounds. There is limited understanding, however, of people’s experiences of stigma, the underlying drivers, intersections with ethnicity, gender, and citizenship status, and how powerful discourses and social institutions create and perpetuate systems of stigma. This review aims to synthesise and critically analyse qualitative evidence to understand how stigma associated with mental health conditions and AOD use operates among people from migrant and ethnic minority groups.

**Methods:**

Qualitative evidence will be identified using MEDLINE, Embase, PsycINFO, CINAHL, Applied Social Sciences Index and Sociological Abstracts. Two reviewers will screen the titles, abstracts and full-text articles. Eligible studies will include original, empirical, peer-reviewed qualitative evidence, published in English since 1990. Studies must examine stigma in relation to mental health conditions, illicit drug use or alcohol consumption among participants who are from migrant and ethnic minority backgrounds. Studies will be critically appraised using the Joanna Briggs Institute Critical Appraisal Checklist for qualitative studies and the level of confidence in the findings will be assessed using Confidence in the Evidence from Reviews of Qualitative research. Data will be analysed using the ‘best fit’ framework synthesis approach, drawing on the Health Stigma and Discrimination Framework.

**Discussion:**

This review will provide an in-depth understanding of the stigma associated with mental health conditions and AOD use among people from migrant and ethnic minority backgrounds. The findings will inform culturally responsive interventions that aim to reduce the negative impact of stigma on individuals, families and communities.

**Systematic review registration:**

PROSPERO CRD42021204057

**Supplementary Information:**

The online version contains supplementary material available at 10.1186/s13643-021-01875-3.

## Introduction

Over the past decade, the global burden of disease from mental health conditions has increased [1]. On average, one in three adults experience a common mental health condition in their lifetime including mood, anxiety and substance use disorders [2]. People from migrant and ethnic minority backgrounds (defined as populations other than the dominant majority of a country in terms of numerical proportions and power positions, particularly migrants, refugees, people seeking asylum and minority groups from non-main English-speaking countries [3]) may be at higher risk of mental health conditions and substance use disorders than the general population [4, 5]. Risk factors include increased experiences of trauma, acculturation stress, and social and economic disadvantage [4, 5]. Research suggests that some young people from refugee backgrounds report heavy alcohol consumption to cope with trauma, boredom and frustration and heightened experience of family conflict, contact with the criminal justice system and homelessness [6]. Evidence also suggests that there are barriers to accessing mental health and alcohol and other drug (AOD) treatment for people from migrant and ethnic minority backgrounds [7] including limited knowledge of where, why or how to seek help, fear of hospitalisation, possible adverse implications for visa applications, limited provision of culturally responsive services, and stigma [8–12].

### Stigma and health

Erving Goffman described stigma as a social process in which a person or group’s attribute, condition or status is identified as different, less desirable or dangerous [13]. People may experience stigma based on physical ability, individual character, health conditions, race, sexual identity, or religion [13]. Illicit drug use and particularly, drug dependence, can be seen as categories which create stigma by their implicit definition of otherness [14]. Since Goffman’s work, there has been a proliferation of stigma research, particularly in relation to health. Link and Phelan conceptualised stigma as the convergence of labelling, stereotyping, separation and discrimination [15]. In this process, differences are identified, labelled as socially important, and associated with negative attributes. Separation occurs when those who are labelled are considered fundamentally different from the norm, which can lead to discrimination or status loss through being devalued, rejected or excluded [15]. Although stigma and discrimination are sometimes used interchangeably, they are distinct: stigma is a broader concept that involves multiple components (including discrimination) whereas discrimination refers to the unequal treatment or constrained opportunities of members of a social group [16]. Discrimination can occur at an individual or structural level where institutional practices and environments create barriers to inclusion. For example, people living with HIV can experience stigma when they are framed as dangerous and immoral, which may result in discrimination in healthcare settings or rejection from social groups [17]. Internalized stigma can also occur when people apply negative stereotypes to themselves and come to expect or fear rejection [15]. Importantly, the components of stigma can only unfold within systems of social, economic and political power that enable the assembly of stigmatising discourses or institutions [15]. Stigma has been shown to negatively affect employment, housing, access to medical care, treatment compliance and help-seeking behaviours [18]. It can also contribute to chronic stress which may increase hypertension and worsen existing medical conditions [18].

### Mental health stigma

Stigma has been examined across a variety of health conditions [14, 15]. People with mental health conditions are often labelled as dangerous, unpredictable, incompetent and dependent on others [19]. Importantly, stigma associated with mental health conditions is shaped by cultural values, norms and beliefs. Three reviews have examined these cultural aspects of stigma; an integrative narrative review exploring mental illness stigma among specific racial and ethnic groups in the USA, a systematic review exploring mental illness stigma among Asian, Black and Latinx Americans and a systematic review identifying meaningful cultural aspects of stigma in non-Western European cultural groups [20–22]. Findings from these reviews suggested that there were similarities and differences in mental illness stigma between racial and ethnic groups. For example, a common finding across groups was that negative labels associated with mental health conditions were often applied to an individual’s family [20–22]. Findings within specific ethnic groups demonstrated the importance of protecting the family reputation from mental illness stigma among Asian communities, the role of historical and current racism in shaping stigma for Black Americans and the perception that mental illness was incongruent with the hardworking values of Latino groups [20–22]. Studies have also documented the negative experiences of stigma for people from migrant and ethnic minority backgrounds including discrimination, prejudice, exclusion and internalised shame [19, 22]. Family members associated with people who have mental health conditions may also be treated unfairly, excluded from social life and feel ashamed [23]. Systematic reviews focused on migrant and ethnic minority populations in the USA and Europe have reported that stigma is a common barrier that prevents access to mental health care [24, 25]. Few studies have examined how stigma associated with mental health conditions intersects with other characteristics or used theory to enhance understanding of the stigma process [22].

### Stigma associated with alcohol and other drug use

Studies have also investigated AOD use stigma and demonstrated that people who inject drugs are often perceived as immoral, irresponsible, undeserving of help, burdensome, deviant and dishonest [26]. Stigma is also evident when people with alcohol dependence are blamed for their ‘voluntary’ condition for ‘choosing’ to use alcohol in ways considered unacceptable by society [27]. Previous qualitative findings have indicated that people with substance dependence experience stigma within their interpersonal relationships, healthcare settings, the criminal justice system, the media and in political and legal systems that criminalise certain types of drug use [14, 28]. Stigma contributes to poor quality healthcare and hinders evidence-based responses such as supervised injecting facilities [26]. People with a dual diagnosis of a mental health condition and substance use disorder may experience higher levels of stigma and discrimination and poorer quality of healthcare than those with a single diagnosis [29].

Existing studies have identified that stigma associated with AOD use is a barrier that impedes access to support for migrant and ethnic minority communities [9]. Qualitative research with young African refugees in Australia identified there were significant risks if they were discovered drinking alcohol including exclusion from their families and broader cultural communities [6]. Similarly, this sample also perceived that injecting drug use was shameful for both the individual and their families [30]. Research with people from Pakistani Muslim backgrounds in Britain identified that alcohol consumption was perceived as dishonourable and incongruent with Islamic values [31]. Gender is also important, with Muslim females at high risk of gossip, reputational damage and being labelled as ‘poor marriage material’ if discovered consuming alcohol [32]. The shame associated with alcohol consumption in some cultures likely encourages people to hide their alcohol consumption to avoid damaging their families reputation [33]. Although some individual studies have investigated stigma associated with AOD use among migrant and ethnic minority groups, to our knowledge, there has been no dedicated effort to bring this body of evidence together. Other reviews that are focused more generally on AOD treatment and health promotion programmes have identified that stigma is a barrier to help-seeking but do not provide in-depth information about the process of stigma and its intersections with other characteristics [34, 35].

### Stigma in the lives of people from migrant and ethnic minority backgrounds

Intersectionality emphasises that multiple facets of people’s identity and social positions interact to shape experiences of stigma and discrimination [36]. Although most existing research focuses on people’s experience of stigma due to one health condition [37], intersectionality has been integrated into various stigma frameworks [38, 39]. In their systematic review, Fox et al. acknowledged that people’s experiences of mental illness stigma differed based on characteristics representative of privilege and marginalisation including their specific diagnosis, socio-economic status, race, culture and gender [39]. Intersectionality is particularly important when considering people with migrant and ethnic minority backgrounds because they often experience discrimination based on multiple identity characteristics [20]. For example, one study showed that Latinx people who injected drugs were perceived as more deserving of punishment than help in comparison to white people who injected drugs [40]. Experiences of mental health and AOD use stigma may also be shaped by migration status. People from migrant and ethnic minority backgrounds are often held to higher standards than others, expected to be upstanding citizens or ‘model minorities’ [41], so that ‘deviant’ behaviour may be perceived more negatively. Further research is needed to understand the nature of the relationship between stigma associated with mental health and AOD use, and discourses and practices intersecting with culture, gender and ethnicity [38].

### Rationale

Stigma is a complex concept involving interrelated components and power systems [42]. Although stigma has been commonly identified as an important barrier to help-seeking among migrant and ethnic minority communities [9, 24, 25], responses to address stigma are hampered by a lack of understanding of people’s experiences, intersectional factors, the underlying drivers and the powerful discourses, institutions and systems that enable stigma to unfold. Research has acknowledged that interventions targeting stigma must address multiple levels including social, policy and legal structures thus incorporating theory that acknowledges these structures is important [28]. Although some reviews have explored cultural factors and mental illness stigma, these have predominantly focused on migrant and ethnic minority groups in the USA [20, 21]. Our review expands the knowledge base by examining global literature and drawing on robust theory to synthesise findings. Our review will also address understudied areas of intersectional stigma and stigma in relation to AOD use. A deeper understanding of the drivers and facilitators, manifestations, intersections, outcomes and impacts of stigma among migrant and ethnic minority populations will guide future research and inform more socially and culturally responsive interventions.

### Guiding theory

Our understanding of stigma in this review draws on Bourdieu’s concepts of symbolic power and Link and Phelan’s concept of stigma power. Bourdieu described the term ‘habitus’ which suggests that people’s beliefs, attitudes, behaviours and knowledge are shaped by life experiences and social positions as defined by important institutions such as religion, race, gender and social class [43]. People develop skills, achievements and a sense of identity that reflect their social group membership, known as cultural capital. These social institutions create power systems that value certain identities over others [43]. Symbolic power refers to the ability to define what constitutes ‘reality’, and impose a legitimate or orthodox version of the social world on others [44]. Stigma is a form of symbolic power because those who articulate orthodox discourses via the social order are in a strong position to determine what is legitimate, valuable and worthy. Bourdieu argues that people unconsciously accept the social hierarchy established via orthodox discourses, which means that symbolic power and associated systems such as stigma can be ‘misrecognised’ as normal, and in some cases, unquestionable, cultural arrangements [45]. Link and Phelan expanded on these ideas and proposed that stigma creates and maintains social hierarchies [46]. Stigma power highlights how culture determines whether certain characteristics are valued or not. People with stigmatised characteristics are generally conscious of the negative labels placed upon them and the risk of being devalued or discriminated against. This awareness increases concern that people should ‘stay in’ to avoid negative cultural evaluation, ‘stay away’ from potentially threatening environments and ‘stay down’ by accepting their lower worth. In this regard, stigma power is a resource that acts to perpetuate existing arrangements of power [46].

**Fig. 1:**
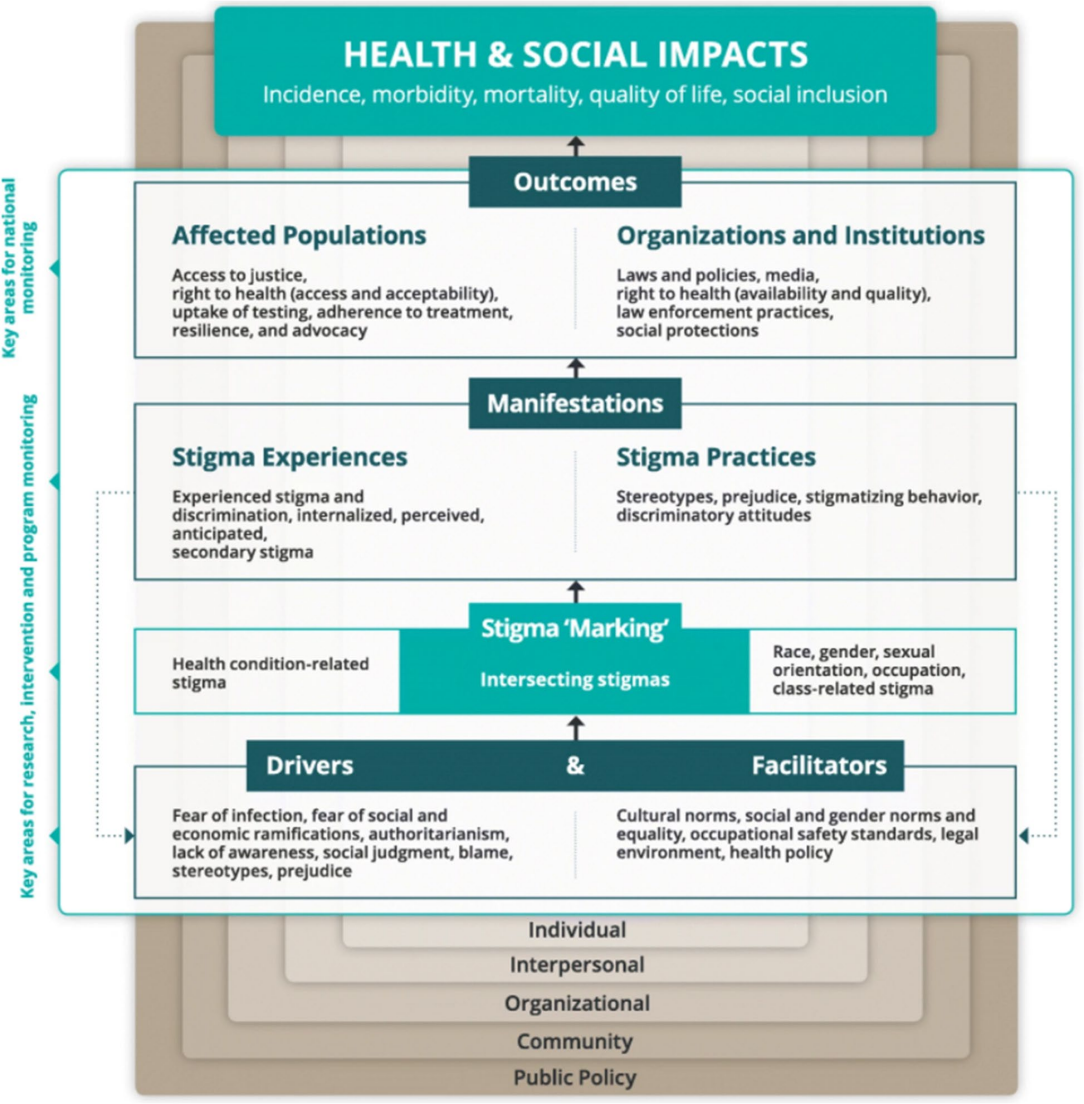
Health Stigma and Discrimination Framework [38]

Our review will be guided by the Health Stigma and Discrimination Framework which acknowledges that multiple domains interact to produce stigma (Fig. 1) [38]. This framework will be used to formulate our research questions, synthesise the data from included studies and identify where gaps in the literature remain and where future interventions could be targeted. Drivers are conceptualised as inherently negative factors that enable stigma (e.g. fear of people with a mental health condition) and facilitators are societal factors that can have positive or negative influences on stigmatisation, for example, cultural and gender norms. The drivers and facilitators of stigma reflect Bourdieu’s idea that within any society, certain cultural attributes are defined as worthy or orthodox—or in some cases unquestionable—while others are devalued. Drivers and facilitators combine to determine whether a person or group are ‘marked’ based on their stigmatised characteristic. ‘Stigma marking’ occurs when stigma is applied to a person or group based on their mental health condition and/or AOD use. This health-related stigma may also intersect with stigma related to other factors such as ethnicity, gender and citizenship status. Once people have been ‘marked’, stigma can manifest as experiences (lived realities such as discrimination and internalised, anticipated, perceived and secondary stigma) and practices (the beliefs, attitudes and actions directed towards a stigmatised person or group). Stigmatisation also leads to ‘outcomes’ for affected populations and organisations (such as access to treatment and support) and impacts on broader health consequences (e.g. quality of life and relationships) [38]. Importantly, the framework explicitly states that stigma is not ‘a thing which individuals impose on others’ but relies on the broader social, cultural, political and economic forces that structure stigma. This reference to systems of power relates to Bourdieu’s concept of symbolic power and Link and Phelan’s description of stigma power.

Figure 1 was originally created by Stangl et al. [38] and has been reproduced under the terms of the Creative Commons Attribution 4.0 International License (https://creativecommons.org/licenses/by/4.0/) which permits unrestricted use, distribution, and reproduction in any medium with appropriate credit. No changes were made to this figure.

### Objectives

The objectives of this review are to identify, synthesise and critically analyse qualitative evidence exploring stigma among people from migrant and ethnic minority backgrounds in relation to mental health conditions, alcohol consumption and illicit drug use. It will also explore the drivers and facilitators underlying stigma, intersecting factors and how these combine to impact relationships, help-seeking and access to support services. The specific review questions include:

1. What are the underlying drivers and facilitators of stigma associated with mental health conditions and/or AOD use among migrant and ethnic minority groups?
2. How does stigma associated with mental health conditions and/or AOD use intersect with other stigmatised characteristics?
3. How does stigma associated with mental health conditions and/or AOD use manifest as experiences and practices among people from migrant and ethnic minority backgrounds?
4. What are the outcomes and impacts of stigma associated with mental health conditions and/or AOD use for people from migrant and ethnic minority backgrounds?

## Methods

This review protocol has been reported in accordance with the Preferred Reporting Items for Systematic Reviews and Meta-analysis Protocols (PRISMA-P) checklist [47] (Additional File 1) and the Enhancing transparency in reporting the synthesis of qualitative research checklist [48].

### Eligibility criteria

This review uses the Sample, Phenomenon of Interest, Design, Evaluation, Research type (SPIDER) tool to guide the search strategy and eligibility criteria [49].

1. *Sample*: This review will include studies where results have been reported for participants who self-identify or have been categorised as a migrant or ethnic minority in low, middle or high-income countries. Studies can include people from migrant and ethnic minority backgrounds who are personally experiencing mental health conditions or using alcohol and/or other drugs, general community members, caregivers or family members. Studies that include a broader study sample (e.g. service providers or non-migrant and ethnic minority groups) will be included if the results for migrant and ethnic minority participants are reported separately or can be distinguished in the results. Although there is no universally agreed upon term, our sample draws upon definitions from the International Glossary on Migration [50] to define the population. We use the umbrella term ‘migrant and ethnic minority’ to encompass the following groups recognising that there is both overlap and important differences between the terms.
  Migrant: A person who has moved away from their usual residence whether within a country or across an international border, temporarily or permanently and for a variety of reasons. We focus on migrants from non-main English-speaking countries.
  Minority: A group with fewer numbers compared to the broader population in a non-dominant and less powerful position whose members possess ethnic, religious or linguistic characteristics differing from those of the dominant population.
  Refugee: A person who, owing to a well-founded fear of persecution for reasons of race, religion, nationality, membership of a particular social group or political opinion is outside the country of their nationality and is unable or, owing to such fear, is unwilling to avail themselves of the protection of that country; or who, not having a nationality and being outside the country of their former habitual residents as a result of such events, is unable or, owing to such fear, is unwilling to return to it
  Person seeking asylum: An individual who is seeking international protection.
  International students and other temporary migrants
2. *Phenomenon of interest*: Studies must explore stigma in relation to mental health conditions, alcohol consumption or illicit drug use (at any level including recreational, problematic or harmful use and dependence). Stigma must be identified as an aim, research question, key theme or major finding in the results
3. *Design*: Methodologies such as ethnography, phenomenology, action research or community-based participatory research and/or data collection techniques including focus groups and interviews
4. *Evaluation*: Perceptions and experiences related to the drivers and facilitators of stigma, intersecting factors, stigma manifestations, outcomes and impacts
5. *Research type*: Original peer-reviewed qualitative studies or other study designs (e.g. mixed methods or evaluation research) with relevant qualitative components published in English from 1990 to present.

The following studies will be excluded from our review:

- Quantitative studies
- Content, document or policy analyses
- Abstracts, conference presentations, dissertations, systematic reviews, literature reviews and commentaries
- Published in language(s) other than English
- Studies that focus on Indigenous or First Nation’s people (e.g. people who identify as Aboriginal and/or Torres Strait Islander). These groups have unique experiences underpinned by histories of colonisation, dispossession, and discrimination; we do not feel that we can do justice to these populations within the context of this review
- Studies that focus on migrants from main English-speaking countries who do not identify with an ethnic minority group and are less likely to experience power disparities with the dominant population of a country
- Studies where participants are health professionals, mental health or AOD service providers from migrant and ethnic minority backgrounds
- Studies that mention stigma but do not explore the topic in-depth
- Studies that focus on tobacco, medicinal cannabis or prescription medication use
- Grey literature

### Information sources

Original peer-reviewed articles will be identified through the databases MEDLINE, Embase, PsycINFO, CINAHL, Applied Social Sciences Index and Sociological Abstracts to capture citations from a broad range of disciplines. Reference lists of included studies will be checked and well-known researchers in the field of stigma will be contacted for additional relevant articles. Studies will be restricted to English language articles published from 1990 to current. The search will be re-run prior to the final synthesis.

### Search strategy

The search strategy will include a combination of MeSH terms and key words to capture the SPIDER parameters: (Sample: migrant and ethnic minority) AND (phenomenon 1: mental health OR phenomenon 2: AOD use) AND (phenomenon 3: stigma) AND (research type or design: qualitative studies, frameworks or methods) (see Additional File 2). Search terms for each database will be developed and refined with the assistance of a librarian.

### Study records

#### Data management

Records will be downloaded from each database into EndNote and uploaded to Covidence where duplicates will be removed. Titles, abstracts and full-text articles will be screened and reviewed in Covidence. Full-text articles will be imported into Dedoose for coding and synthesis (Dedoose Version 9.0.17, (2021). Los Angeles, CA: SocioCultural Research Consultants, LLC; www.dedoose.com).

#### Selection process

The selection of articles to be included in the review will be managed using Covidence. Titles and abstracts will be reviewed by two independent reviewers using pre-determined inclusion and exclusion criteria. Full-text articles that meet inclusion criteria based on title and abstract screening will be reviewed for eligibility by two independent reviewers. Any disagreement will be resolved through discussion and consensus or through discussion with a third reviewer where necessary. The Preferred Reporting Items for Systematic Reviews and Meta-analysis (PRISMA) flowchart will be used to document the number of studies identified, included and excluded throughout the selection process.

#### Data collection process and data items

Using Covidence, one reviewer will extract study characteristics from each included article using a data extraction template. The template will be pilot tested on five studies to determine items to include. Planned items for extraction are author, year of publication, country, population, participant characteristics, research questions, data collection, methodology, theoretical frameworks and type of analysis. A second reviewer will check all extracted data against the full-text articles and note any errors to be updated by the first reviewer.

#### Critical appraisal of individual studies

Two reviewers will independently appraise the methodological quality of each included study using the Joanna Briggs Institute critical appraisal checklist for qualitative research [51]. The checklist includes ten questions to determine whether there is congruity between the research methodology with the philosophical perspective, research questions, data collection, representation and analysis of data and interpretation of results. It also contains questions related to the researchers’ theoretical position, influence of the researcher, representation of participant voices, ethics and conclusions. Discrepancies will be resolved through discussion and consensus or involvement of a third reviewer where required. The tool will be used to determine if studies are rated as low, medium or high. We will not exclude articles from the review based on their rating.

#### Data synthesis

Data will be synthesised using the ‘best-fit’ framework synthesis approach [52, 53]. This approach is well suited to qualitative reviews where a suitable theoretical framework already exists. The approach involves coding data to the selected framework, in our case the Health Stigma and Discrimination Framework [38], then using an inductive thematic analysis to code data which cannot be accommodated within the existing framework [52, 53]. Drawing on the Health Stigma and Discrimination Framework and our knowledge of the literature, we have mapped out key concepts that we hypothesise will be relevant in understanding stigma among people from migrant and ethnic minority backgrounds in relation to mental health conditions and AOD use (Table 1).

**Table 1:** Applying the Health Stigma and Discrimination Framework to this review

Domain	Sub-domain	Topic codes
**Drivers and Facilitators**	**Drivers**	• Fear • Poor knowledge/awareness • Prejudice • Stereotypes
	**Facilitators**	• Social, gender, religious and cultural norms and beliefs which determine whether alcohol consumption or illicit drug use are acceptable in particular settings • Laws and policies
**Health condition related stigma**	**Intersecting fields of stigma**	• Ethnicity, gender, sexual identity, citizenship status, socio-economic status, age, other health conditions
**Stigma manifestations**	**Experiences**	• Internalised stigma (people feeling shame and personally taking on the negative labels associated with AOD use and mental health conditions) • Experienced stigma (verbal abuse, vilification) • Anticipated stigma (expectation or fear of bias if others discover their AOD use or mental health condition) • Secondary stigma (negative labels applied to family and friends) • Experienced discrimination (unfair treatment or constrained opportunities)
	**Practices**	• Being stereotyped by members of the public or service providers • Stigmatising behaviours (exclusion, avoidance, rejection, gossip) • Discriminatory attitudes • Expressions of prejudice
**Outcomes**	**Individual**	• Concealment or non-disclosure • Increase in risk behaviours • Limited ability to seek and obtain access to appropriate mental health and AOD services • Informal help-seeking • Resilience and advocacy through rejection of stereotypes
	**Organisations and institutions**	• Responses or interventions that can be implemented at an organisational or institutional level to address stigma
**Impacts**	**Impacts**	• Reduced quality of life • Increased isolation and loneliness • Decreased participation in employment and housing • Increased contact with the criminal justice system • Exacerbation of existing mental health conditions • Increase in depression, anxiety and social isolation • Long-term break down in relationships

Two reviewers will pilot test the framework on five included studies and refine sub-domains, topic codes and definitions through discussion. One reviewer will then apply the framework to each included article by highlighting relevant sections of the results and coding the data to the topic codes, sub-domains and domains outlined in Table 1. Data that will be coded include direct quotes from participants and the primary authors’ description of results given that their interpretation is generally supported by additional data and contextual information [54, 55]. In articles that include additional participants who do not meet the inclusion criteria, only data from migrant and ethnic minority participants will be coded. A second reviewer will code one quarter of the articles to ensure the framework is consistently applied; any discrepancies will be resolved through discussion or consultation with a third reviewer. Data that do not fit the framework, will be assigned to an ‘other’ code; these excerpts will be reviewed, inductively coded and presented in an adapted version of the framework [52, 53].

Throughout the coding process, reviewers will create memos to record their insights on the data and document links between codes. One reviewer will create a summary of each article to capture the main findings related to stigma. One reviewer will draw on the coded data, memos and article summaries to synthesise the key findings for each domain of the Health Stigma and Discrimination Framework. All co-authors will reflect upon and refine the synthesised findings, which will then be written as a narrative with supporting quotes from the included studies. Findings will also be visualised through presenting the adapted version of the framework that best fits the included studies. Importantly, data synthesis will be shaped by the positionality of the review team which is made up of academics with backgrounds in AOD, sociology, young people’s health, migrant inclusion and social cohesion based in Australia and Myanmar.

#### Confidence in cumulative evidence

The level of confidence in the findings from this review will be assessed using the Grading of Recommendations Assessment, Development and Evaluation-Confidence in the Evidence from Reviews of Qualitative research (GRADE-CERQual) [56]. This method assesses four components including methodological limitations of included studies, coherence of review findings, adequacy of data that contribute to review findings and relevance of each included study to the review question. Each review finding will be graded as high, moderate, low or very low confidence.

## Discussion

In this paper, we describe the protocol for a systematic review of qualitative studies exploring the stigma associated with AOD use and mental health conditions among migrant and ethnic minority groups. Stigma has been identified as a major barrier to accessing support for AOD use and mental health conditions among migrant and ethnic minority communities [12]. Existing evidence suggests that further research underpinned by theory is needed to understand the intersectional factors that influence AOD use and mental health stigma [20, 21]. Use of best fit framework analysis guided by the Health Stigma and Discrimination Framework will enable us to provide insight into the different domains of stigma, identify gaps in the literature and provide recommendations for future research.

Any important updates to the protocol will be dated and track changed in PROSPERO. Additionally, results from this review will be shared with policy-makers and service providers to inform future policy responses and healthcare delivery. Limitations of this review include the exclusion of studies published in languages other than English. We also acknowledge that qualitative systematic reviews involve taking findings out of their original context and synthesising data to answer new research questions, which is particularly complex when studies include multiple countries and cultures [54]. Importantly, the evidence generated from this review will be used to advocate for culturally responsive interventions that aim to reduce the negative outcomes of stigma for individuals, families and communities.

## Supplementary Information

**Additional file 1.** PRISMA checklist.

**Additional file 2.** Example of search terms for MEDLINE.

## Data Availability

Not applicable

## Abbreviations

GRADE-CERQual: Grading of Recommendations Assessment, Development and Evaluation-Confidence in the Evidence from Reviews of Qualitative research
PRISMA: Preferred Reporting Items for Systematic Reviews and Meta-analysis
PRISMA-P: Preferred Reporting Items for Systematic Reviews and Meta-analysis Protocols
SPIDER: Sample, Phenomenon of Interest, Design, Evaluation, Research type
