# Supplementary material for: Exploring stigma associated with mental health conditions and alcohol and other drug use among people from migrant and ethnic minority backgrounds: a protocol for a systematic review of qualitative studies
Source: Syst Rev. 2022 Jan 18;11:12. doi: 10.1186/s13643-021-01875-3 (PMC8767730; doi:10.1186/s13643-021-01875-3)
Supplement: Supplementary file 2 — Additional file 2. Example of search terms for MEDLINE. [file 13643_2021_1875_MOESM2_ESM.pdf]

Database(s): **Ovid MEDLINE(R)** 1946 to November 13, 2020

Search Strategy:

#	Searches	Results
1	african continental ancestry group/	38005
2	asian continental ancestry group/	66201
3	ethnic groups/ or african americans/ or arabs/ or asian americans/ or hispanic americans/	140478
4	acculturation/	6466
5	"emigrants and immigrants"/	12490
6	Minority Groups/	14183
7	"Transients and Migrants"/	11887
8	Refugees/	10469
9	Refugee Camps/	171
10	cultural diversity/	11619
11	Human Migration/	1213
12	undocumented immigrants/	375
13	CALD.mp.	293
14	(transient* or migrant* or immigrant* or emigrant* or refugee* or migration).mp.	578751
15	(asylum adj2 seek*).mp.	1633
16	((ethnic* or racial) adj (minor* or group* or population* or communit* or origin* or people* or background*)).mp.	102563
17	(cultur* adj3 divers*).mp.	15976
18	(displac* adj1 (internal* or forced or mass or person* or people* or population*)).mp.	1675
19	or/1-18	842153
20	Mental Health/	39981
21	Mental Disorders/	164004
22	Mentally Ill Persons/	6203
23	Anxiety/	82879
24	anxiety disorders/ or agoraphobia/ or anxiety, separation/ or neurotic disorders/ or obsessive-compulsive disorder/ or hoarding disorder/ or panic disorder/ or phobic disorders/ or phobia, social/	78786
25	depression/ or mood disorders/ or depressive disorder/ or depression, postpartum/ or depressive disorder, major/ or depressive disorder, treatment-resistant/ or dysthymic disorder/ or premenstrual dysphoric disorder/ or seasonal affective disorder/ or cyclothymic disorder/	231443
26	stress disorders, traumatic/ or psychological trauma/ or historical trauma/ or stress disorders, post-traumatic/ or stress disorders, traumatic, acute/	35154
27	self-injurious behavior/ or self mutilation/ or suicide/	49241
28	schizophrenia/ or schizophrenia.mp.	130428
29	Psychology, Clinical/ or stress, psychological/	125127
30	mental health services/ or community mental health services/ or counseling/ or social work, psychiatric/	89374
31	mental health recovery/ or psychiatric rehabilitation/	537
32	(mental* adj1 (health* or ill* or well* or disease* or disorder* or problem* or condition* or issue*)).mp.	332694
33	(depress* or anxiet* or trauma*).mp.	959528
34	Psychotic Disorders/ or psychotic.mp. or psychosis.mp.	77276
35	(PTSD or posttrauma* or post?trauma* or post trauma*).mp.	70604
36	(affective disorder? or dysthymi* or agoraphobi* or panic).mp.	35867
37	"Diagnosis, Dual (Psychiatry)"/ or dual diagnos*.mp.	4501
38	or/20-37	1495507
39	substance-related disorders/ or alcohol-related disorders/ or alcohol-induced disorders/ or psychoses, alcoholic/ or alcoholic intoxication/ or alcoholism/ or amphetamine-related disorders/ or cocaine-related disorders/ or drug overdose/ or inhalant abuse/ or marijuana abuse/ or narcotic-related disorders/ or opioid-related disorders/ or heroin dependence/ or morphine dependence/ or opium dependence/ or phencyclidine abuse/ or psychoses, substance-induced/ or substance abuse, intravenous/ or substance abuse, oral/	237964
40	drinking behavior/ or alcohol abstinence/ or alcohol drinking/ or binge drinking/ or alcohol drinking in college/ or underage drinking/	76441
41	Drug-Seeking Behavior/	1360
42	Drug Users/	3226
43	illicit drugs/	11301
44	Substance Abuse Treatment Centers/	5304
45	Alcoholics/	850
46	crack cocaine/	1432
47	amphetamines/ or methamphetamine/	15653
48	(PWID or PWUD).mp.	1540
49	(cannabis or marijuana).mp.	29422

50	(heroin or opiate* or opium or opioid* or narcotic* or cocaine).mp.	200778
51	AOD.mp.	1542
52	(drug adj2 (illicit* or illegal* or inject*)).mp.	21237
53	((drug* or substance* or alcohol*) adj2 (addict* or dependen* or problem* or abuse* or treat*)).mp.	191879
54	alcoholic beverages/ or absinthe/ or beer/ or wine/	20399
55	(alcohol* adj (drink* or consum*)).mp.	89421
56	(khat or qat).mp.	802
57	(amphetamine* or methamphetamine*).mp.	41107
58	(ecstasy or mdma or ketamine or hallucinogen* or inhalant*).mp.	32478
59	or/39-58	624964
60	38 or 59	2004935
61	dehumanization/ or prejudice/ or social discrimination/ or social distance/ or social marginalization/ or social stigma/ or stereotyping/ or stereotyped behavior/	53540
62	social values/	19855
63	Taboo/	836
64	rejection, psychology/ or scapegoating/	1850
65	stigma*.mp.	32548
66	stereotyp*.mp.	34876
67	prejudic*.mp.	28375
68	(social* distanc* or ostraci?*).mp.	5095
69	(dehumanis* or dehumaniz*).mp.	1072
70	(dishonor* or dishonour*).mp.	72
71	((social* or communit* or cultural* or famil* or ethnic* or racial*) adj (exclusion* or exclud*)).mp.	1751
72	(marginalis* or marginaliz*).mp.	6624
73	taboo*.mp.	2450
74	shame*.mp.	5360
75	(discriminat* adj2 social*).mp.	2603
76	or/61-75	125468
77	community-based participatory research/ or grounded theory/ or qualitative research/	62823
78	interview/	28590
79	Interview, Psychological/	15102
80	focus groups/ or interviews as topic/	89619
81	anthropology/ or anthropology, cultural/	9834
82	qualitative*.mp.	243626
83	(phenomenolog* or phenomenograph*).mp.	20571
84	(ethnograph* or ground* theor*).mp.	19380
85	interview*.mp.	346563
86	(focus group* or group* discuss*).mp.	47966
87	lived experience*.mp.	4940
88	(thematic* or theme$).mp.	93078
89	((participatory adj2 (study or research or project)) or photovoice* or photo?voice).mp.	7495
90	narration/ or narration.mp.	8903
91	mix* method*.mp.	17186
92	or/77-91	602173
93	19 and 60 and 76 and 92	1134
94	limit 93 to english language	1112
95	limit 94 to yr="1990 -Current"	1083

Result 1.

**Unique Identifier**	31955360
**Title**	The Effect of **Depression** on Adherence to HIV Pre-exposure Prophylaxis Among High-Risk South African Women in HPTN 067/ADAPT.
**Source**	AIDS & Behavior. 24(7):2178-2187, 2020 Jul.
**Version ID**	1
